# COVID-19 Whole-Genome Resequencing with Redundant Tiling PCR and Subtract-Based Amplicon Normalization Successfully Characterized SARS-CoV-2 Variants in Clinical Specimens

**DOI:** 10.1155/2022/2109641

**Published:** 2022-09-28

**Authors:** Tatsuki Sugi, Mizanur Rahman, Rummana Rahim, Abu Hasan, Naoko Kawai, Kyoko Hayashida, Junya Yamagishi

**Affiliations:** ^1^Division of Collaboration and Education, International Institute for Zoonosis Control, Hokkaido University, Nishi10-Kita20, Sapporo, Japan; ^2^Evercare Hospital Dhaka, Bashundhara R/A, Block-E, Plot-81, Dhaka-1229, Dhaka, Bangladesh; ^3^International Collaboration Unit, International Institute for Zoonosis Control, Hokkaido University, Nishi10-Kita20, Sapporo, Japan

## Abstract

With an increasing number of severe acute respiratory syndrome coronavirus-2 (SARS-CoV-2) sequences gathered worldwide, we recognize that deletion mutants and nucleotide substitutions that may affect whole-genome sequencing are accumulating. Here, we propose an additional strategy for tiling PCR for whole-genome resequencing, which can make the pipeline robust for mutations at the primer annealing site by a redundant amplicon scheme. We further demonstrated that subtracting overrepresented amplicons from the multiplex PCR products reduced the bias of the next-generation sequencing (NGS) library, resulting in decreasing required sequencing reads per sample. We applied this sequencing strategy to clinical specimens collected in Bangladesh. More than 80% out of the 304 samples were successfully sequenced. Less than 5% were ambiguous nucleotides, and several known variants were detected. With the additional strategies presented here, we believe that whole-genome resequencing of SARS-CoV-2 from clinical samples can be optimized.

## 1. Introduction

Whole-genome sequencing data is an essential resource for coronavirus disease 2019 (COVID-19) genome epidemiology. Currently, an increasing amount of sequence data is collected worldwide, with up to 8,941,854 records as of March 18, 2022 (https://www.covid19dataportal.org/). These sequencing data were used to estimate the infection path or virus evolution [1] and how the virus was transmitted at the early phase of the outbreak to aid public health decision-making [2]. Furthermore, they have been used to track viral lineages circulating globally to detect the emergence of variants of concern with the increased reproduction rate, increased virulence, or decreased effectiveness of control measures such as vaccines (https://www.who.int/en/activities/tracking-SARS-CoV-2-variants/). The Global Initiative on Sharing All Influenza Data (GISAID) submission tracker [3] shows that the coverage of the sequences is dependent on the region (Africa, 0.75%; Asia, 0.55%; EU, 2.63%; North America, 3.44%; Oceania, 2.15%; and South America, 0.37% (% sequence shared in GISAID with complete dates per total reported cases as of March 20, 2022)), possibly reflecting overall sequencing capacity (reviewed in [4]). Thus, reducing the cost and the required sequencing capacity for determining the whole-genome sequence will help decrease regional bias in sequence coverage.

The current whole-genome sequencing paradigm relies on tiling PCR (ex. ARTIC network: nCoV-2019 sequencing  protocol V.1,  https://dx.doi.org/10.17504/protocols.io.bbmuik6w, which amplifies the whole SARS-CoV-2 genome with at least two sets of multiplex PCR. One of the challenges in tiling PCR-based whole-genome sequencing is amplification bias, especially in clinical specimens with low viral titers [5]. One approach for nonbiased amplification is to use tiling PCR with long amplicons [6, 7]. However, the long amplicon strategy also suffers from low genome coverage due to low virus titers from collected samples [8]. Another strategy is to optimize primer concentration during multiplex PCR. Increasing the concentration of primers in poorly amplified regions partially solved this issue, as reported previously [8]. Another solution to this is redesigning the primers, which showed a promising increase in genome coverage and variant calling efficiency [9, 10].

Another challenge is when viral genome mutations are present at primer annealing sites. Amplicon dropouts due to single nucleotide variations (SNVs) have been reported in the original Artic primer schemes for virus variants of concern, and primer schemes have been updated to address this problem (https://community.artic.network/t/SARS-CoV-2-version-4-scheme-release/312). Using this version, actual genome coverage has been improved [10]. Along with SNVs, amplicon dropouts due to the long deletion variants of virus genomes have been reported [11, 12]. In such a case, the genome region covered only by the dropout amplicon will be lost from the sequence data, so additional experiments should be performed to complete the virus genome sequence.

In this study, two additional strategies which can be combined with the tiling PCR resequencing are proposed. The first strategy is to rescue the amplicon drops caused by the mutation on the primer annealing site by the redundant amplicon scheme. The second strategy is to increase the sequencing depth in the amplicon region with low amplification efficiency by the subtraction of overrepresented amplicons. To establish the proof of concept for these two strategies, we established a new tiling PCR primer scheme with redundancies covering every position of the whole genome by at least two amplicons, except for the 5′- and 3′-ends. We also combined this primer scheme with the subtraction of overrepresented amplicons to reduce bias in the sequenced library. Finally, we validated this sequencing pipeline in clinical samples from Bangladesh and analyzed the viral variants in the cohort.

## 2. Materials and Methods

### 2.1. Clinical Specimens

Nasopharyngeal specimens were collected from 304 patients for diagnosis, and the remaining isolated RNAs were used in this study. The RNA was purified using an MagMAX Viral/Pathogen Nucleic Acid Isolation Kit (Thermo Fisher) according to the manufacturer's instructions. First-strand cDNA was synthesized using a ProtoScript II first-strand cDNA synthesis kit (E6560S, NEB) using oligo-dT and random 9-mer primers. An in vitro amplified virus isolate (EPI_ISL_408667) (labeled NIID) was used as the control virus [13]. The RNA of the control virus was reverse-transcribed with a SuperScript IV system (Thermo Fisher) using oligo-dT and random 6-mer primers.

### 2.2. Redundant Tiling PCR

Redundant tiling PCR was performed using three separate multiplex PCR runs per sample. The primer sets for each multiplex PCR group (#1, #2, and #3) were mixed at a total concentration of 10 *μ*M, and the forward primer was mixed with a biotinylated forward primer with the same sequence at a specified concentration (Supplementary Table 1). For each reaction, 1.2 *μ*L 10× diluted cDNA template, 1.44 *μ*L primer mix, 5 *μ*L Q5 hot start 2× master mix (NEB), and 2.36 *μ*L nuclease-free water were mixed and amplified with the following thermocycling conditions: 98°C for 30 s, 35 cycles of 95°C for 15 s, 63°C for 5 min, and holding indefinitely at 4°C. The PCR products of the three multiplex PCR groups for each specimen were pooled and used for NGS library preparation.

### 2.3. Subtraction of Overamplified Amplicons

Biotinylated PCR amplicons were subtracted from multiplex PCR products as follows. MyOne Streptavidin C1 magnetic beads (13 µL; Thermo Fisher) were washed and resuspended in 13 *μ*L of binding buffer (1 M NaCl, 5 mM Tris-HCl (pH 7.5), 0.5 mM EDTA) and mixed with 13 *μ*L of pooled tiling PCR amplicons. For “without subtraction” control experiments, 13 *μ*L of binding buffer without magnetic beads was mixed with 13 *μ*L of pooled tiling PCR amplicon. After incubation for 1 h at room temperature, the unbound DNA in the supernatant was transferred to a new tube and cleaned with AMPure XP beads (Beckman Coulter) for further NGS library preparation.

### 2.4. Library Preparation and Sequencing: Nanopore Sequencing

Pooled and purified tiling PCR products were used to prepare the Nanopore sequencing library using a ligation sequencing kit (Oxford Nanopore Tech: SQK-LSK110) according to the manufacturer's instructions. The libraries were sequenced using a Flongle Flow Cell (Oxford Nanopore Tech: FLO_FLG001). An amplicon pool for each sample was sequenced with an individual Flongle Flow Cell. Base-calling with Guppy version 4.4.2 (Oxford Nanopore Tech) using a high-accuracy model was performed to obtain FASTQ files.

### 2.5. Library Preparation and Sequencing: Illumina MiSeq

Pooled and purified tiling PCR products were used for sequence library preparation, as described previously [11]. Briefly, after introducing the index sequence for each sample via PCR, all 304 samples were pooled, and pooled libraries of approximately 500 bp were purified via agarose gel electrophoresis. MiSeq 300 bp paired-end sequencing was performed using the MiSeq Reagent Kit V3 (Illumina).

### 2.6. Data Analysis

Nanopore sequences were used to characterize the amplicon bias and read depth for each genome position. Analysis tools were used with default parameters unless otherwise mentioned. Raw reads were preprocessed with duplex_tools version 0.2.7 (Oxford Nanopore Tech) and Porechop version 0.2.4 (https://github.com/rrwick/Porechop) with an adapter threshold = 95 to remove duplex reads and trim adapter sequences. For quality filtering, seqkit [14] was used to select sequences with minimal average quality over 7 and lengths greater than 80 bp but less than 700 bp. Preprocessed sequences were aligned to a reference sequence (Wuhan-Hu-1 GenBank ID: MN908947.3) using minimap2 version 2.22 [15]. After mapping, the primer sequences for amplification were trimmed using iVar version 1.3.1 with the following parameters: -q, 0; -m, 80; -s, 50 [16]. The resulting bam file was further filtered to remove ambiguous reads that could not be assigned to the unique amplicon regions. To calculate the sequencing depth at each genomic position, IGVtools [17] was used to pile up the nucleotides called at each position, and the A, T, G, C, N, and deletion counts were summed as the depth. Normalization was conducted to obtain the depth or count value per 100 kbp mapped reads.

For the Illumina sequencing data, the following pipeline was used for variant detection in the clinical sample, as previously described [11]. The primer sequences were trimmed using pTrimmer version 1.3.3 [18]. Sequences with low-quality scores were also trimmed using Trimmomatic version 0.36 with the parameters LEADING: 20, TRAILING: 20, SLIDINGWINDOW:4:20, and MINLEN:150 [19]. The qualified reads were aligned using minimap2 version 2.17 on the reference sequence. Sequence variants were called using GATK HaplotypeCaller version V4.2.0.0 [20] and then filtered using an in-house script if the population of the polymorphism was less than 0.8 and the number of aligned reads was less than three. The tentative genome sequence for each sample was reconstituted using GATK FastaAlternateReferenceMaker using the filtered VCF dataset. Long deletions and insertions were manually corrected using IGV [17]. To polish the tentative genome sequences, the qualified Illumina reads were aligned using minimap2, and variants were called using GATK HaplotypeCaller, as described above. Finally, low-reliability sequences were masked by N-base. Briefly, read depths were obtained using the SAMtools depth command version 1.12 [21]. Sequences supported by less than five coverages were excluded, while sequences with an alternative genotype and a population of more than 0.3 were also masked. The clades, SNVs, insertions, and deletions were annotated using the Nextclade web service [22]. The uniqueness of the specified SNVs was examined using a dataset provided by RCoV19 [23].

### 2.7. Downsampling

To simulate decreased sequencing reads per sample, the FASTQ raw MiSeq sequencing reads were randomly sampled to give 50% or 25% of total reads in downsampled fastq files. Genome coverages for each sample were determined as described above in the Data Analysis section using those downsampled fastq files.

### 2.8. Ethical Approval

The study was approved by the Evercare Hospital Dhaka and Hokkaido University Ethical Committee (ERC 26/2020-2 and Jinjyu 2020-10, respectively).

## 3. Results

### 3.1. The Redundant Amplicon Scheme Is Robust on Mutations on the Primer Annealing Site, and Amplification Bias was Consistent among Samples

**Figure 1:**
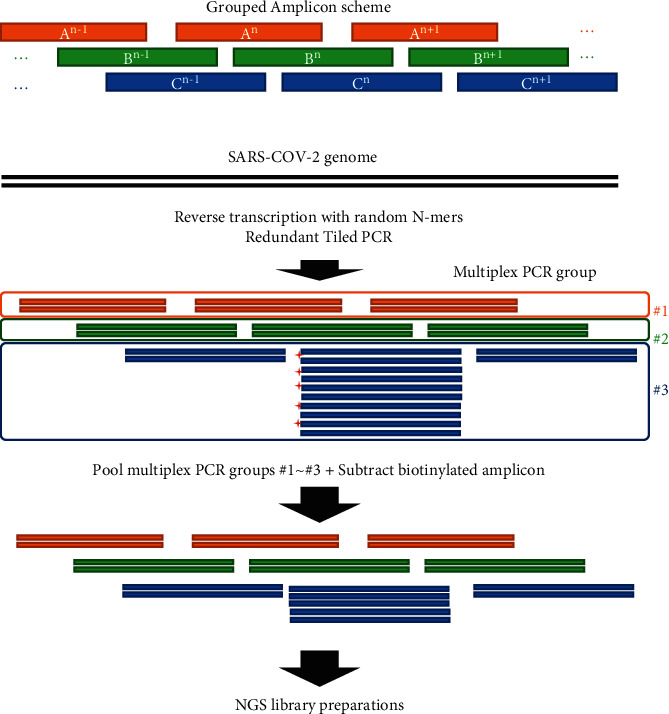
Workflow of the redundant tiling PCR. A schematic illustration of the redundant tiling PCR strategy for the whole-genome sequencing of SARS-CoV-2 is shown. Multiplex PCR was performed for three groups (orange, green, and blue amplicons) with no overlaps within each multiplex PCR. The primers used for this scheme are listed in Supplementary Table 1. After the first cDNA strand was synthesized via reverse transcription, it was subjected to tiling PCR. Using prior knowledge, consistently overrepresented amplicons were marked with biotin using biotinylated primers (red crossed marks) and subtracted from the multiplex PCR products via streptavidin-dependent subtraction. The subtracted multiplex PCR products were then used for NGS library preparation.

**Figure 2:**
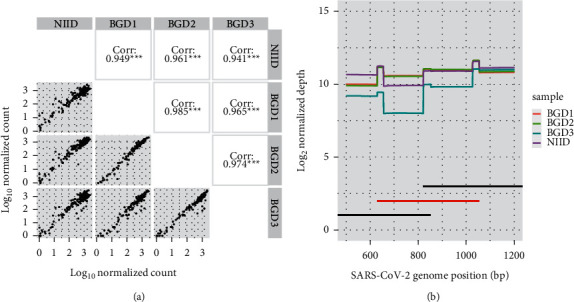
Amplicon bias among four samples is largely consistent but influenced by SNVs on the primer annealing site. (a) Comparison of normalized counts for each amplicon from NIID and three Bangladesh clinical samples (BGD1, 2, and 3). Log10-transformed amplicon counts normalized from 100,000 mapped reads are shown. Pairwise comparisons with Pearson's correlation values are shown in the upper right quadrant. (b) Read depths for each sample in the region spanning region_002 (left black line), region_003 (middle red line), and region_004 (right black line) are shown. The SARS-CoV-2 sequence from BGD3 had a mutation on the reverse primer for region_003. Read depths were normalized from 100,000 mapped reads.

The first concept of our robust whole-genome sequencing strategy is redundancy. We designed redundant primer schemes, and the resulting 147 amplicons enabled the coverage of every position on the SARS-CoV-2 whole genome with at least two amplicon sets, except the 5′- and 3′-ends (Figure 1, Supplementary Table 1). To determine amplification bias throughout the amplicons among different biological samples, we amplified the whole SARS-CoV-2 genome with four samples, comprising one virus isolate (NIID: EPI_ISL_408667) and three samples from patients with COVID-19 (EPI_ISL_11811634, EPI_ISL_11811733, and EPI_ISL_11811834). Log-transformed normalized amplicon counts showed a Pearson correlation coefficient of >0.9 for all comparisons (Figure 2(a)). This suggests that the amplification bias, i.e., the overamplified and poorly amplified regions, is largely consistent among samples. The difference between the reverse transcription methods in NIID and other samples should be noted. The Pearson correlation coefficients of the normalized amplicon counts among three clinical samples (0.985, 0.965 and 0.974 for BGD1 vs. BGD2, BGD1 vs. BGD3, and BGD2 vs. BGD3, respectively) were greater than those between NIID and other samples (0.949, 9.961, and 0.941 for NIID vs. BGD1, BGD2, and BGD3, respectively) (Figure 2(a)). This suggests that reverse transcription methods could be one of the factors which affect amplification biases. When we analyzed the SNVs detected in the primer annealing sites for these four samples, one sample (BGD3) had a nucleotide substitution of C1059T within 5 bp from the 3′-end of primer 003R. The normalized amplicon counts for region_003 (from 630 bp to 1054 bp, without primers) in BGD3 was approximately seven to eleven-fold lower than the other samples without SNVs (898, 1419, 1397, and 126 for NIID, BGD1, BGD2, and BGD3, respectively). Redundant primer schemes, as shown in Figure 2(b), successfully rescued the reduction of sequencing depth in the region by the neighboring amplicons, resulting in only two-to three-fold reductions in the average depth of 1642, 1923, 1925, and 725 reads for NIID, BGD1, BGD2, and BGD3, respectively. This result supports the concept that redundant amplicon schemes can rescue the dropout amplicons derived from mutations at the primer annealing site.

### 3.2. Targeted Subtraction of Overamplified Regions Using Biotinylated Primers

**Figure 3:**
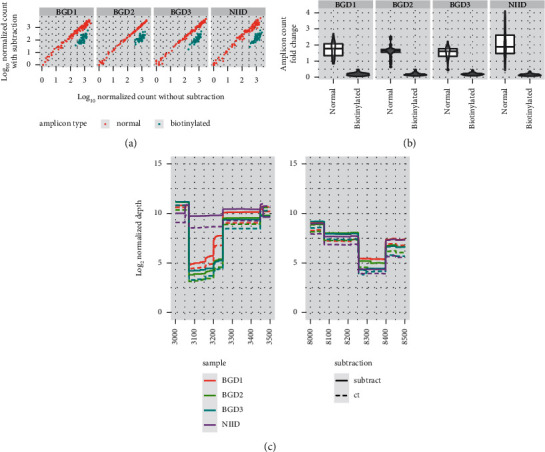
Subtraction of biotinylated amplicons successfully reduced the number of biotinylated amplicons. (a) The NIID, BGD1, 2, and 3 were resequenced with or without biotinylated amplicon subtraction. The read counts for each amplicon region were normalized from 100,000 mapped reads and were log10-transformed. Amplicons with biotinylated primers (blue: biotinylated) and without biotinylated primers (red: normal) are shown for each sample. (b) Fold changes of normalized amplicon counts for each amplicon from the control and subtracted experiments are shown. Distributions of the fold changes are shown in the violin plots, and the 1st quantile, median, and 3^rd^ quantile values are shown in the box plots. Amplicons with biotinylated primers (biotinylated) and without biotinylated primers (normal) are shown for each sample. (c) Normalized read depths for each sample with or without subtraction are shown. The regions with low depth, genome positions 3000–3500 bp (left panel) and 8000–8500 bp (right panel) are also shown. Solid lines: depths for the experiments with subtraction (subtract). Dashed lines: depths for the experiments without subtraction (ct).

The second concept is based on the subtraction of overamplified amplicons (Figure 1). To reduce the overrepresented amplicons from the multiplex PCR products, we introduced a biotinylated primer for the target amplicons (Supplementary Table 1). The tiling PCR products containing biotinylated amplicons were mixed with streptavidin magnetic beads to subtract biotinylated amplicons. Biotinylated amplicons were successfully subtracted from the PCR products from all four samples, independent of the amplicon count (Figures 3(a) and 3(b)). This indicates that subtracting overrepresented amplicons (target amplicons) increased the relative presence of nontarget amplicons with low amplification efficiency. This improvement was confirmed by an increase in the sequencing depth per 100 k normalized mapped reads in regions with low amplification efficiency (3000–3500 bp and 8000–8500 bp) (Figure 3(c)).

### 3.3. Application of the Proposed Resequencing Workflow Using Clinical Specimens from Bangladesh

**Figure 4:**
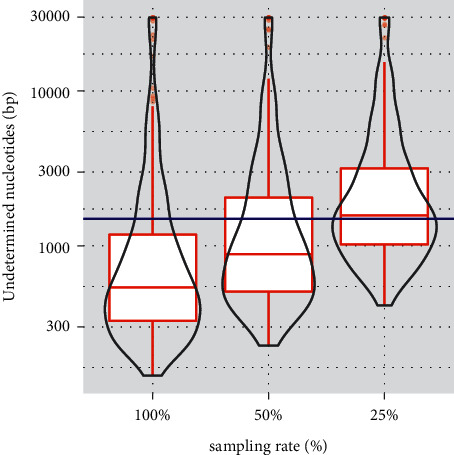
Distribution of undetermined nucleotides length in 304 clinical samples. Undetermined nucleotides with read depths less than five are analyzed using sequence reads from 25% downsampled, 50% downsampled, or whole-FASTQ data (100%) obtained from single MiSeq runs for the 304 samples. Distributions of the lengths of the undetermined nucleotides are shown in the violin plots, while the box plots show the 1^st^ quantile, median, and 3^rd^ quantile values. The blue line shows the threshold of 1495 bp, which is 5% of the whole SARS-CoV-2 genome sequence.

**Figure 5:**
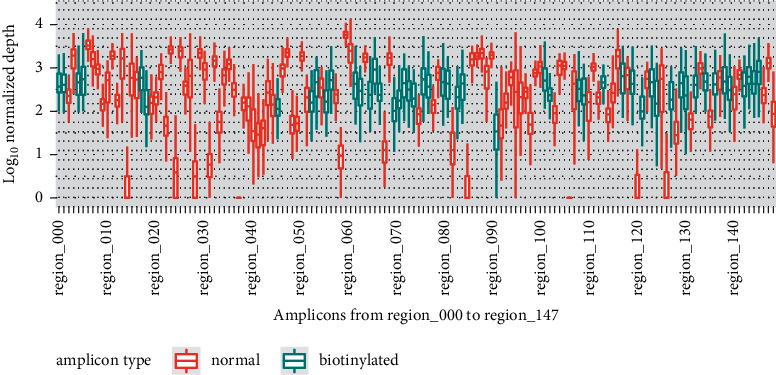
Genome-wide amplicon bias in the 304 clinical samples. Distributions of counts for each amplicon from region_000 to region_147 among the 304 clinical samples are shown. Read numbers per 100,000 mapped reads were log10-transformed to calculate normalized count values. Amplicons with biotinylated primers are marked in blue (biotinylated). Amplicons without biotinylated primers are shown in red (normal). For each distribution, the box plot shows the 1^st^ quantile, median, and 3^rd^ quantile values.

We validated our resequencing workflow using 304 clinical specimens collected in Bangladesh between May 2019 and April 2020. Multiplex sequencing for all samples was executed simultaneously using MiSeq, and 8,368,026 paired reads were obtained. Using this workflow, we successfully acquired 294 whole-genome sequences and deposited them in the GISAID database. The median length of undetermined nucleotides was 542 bp (i.e., 98.2% of the whole genome was successfully covered), including the 95 bp long-terminal regions that were not covered by amplicons (Figure 4). We simulated the effect of sequencing depth on undetermined nucleotides using the downsampling method in our reads. This resulted in a median of 887 and 1577 bp of undetermined nucleotides (i.e., 97.0% and 94.7% coverage, respectively) using 50% and 25% of the reads, respectively (Figure 4), suggesting the potential applicability of increased multiplexing in one MiSeq run. We also validated the biases of each amplicon in the total sequencing library after the subtraction (Figure 5). Some amplicons were difficult to amplify, such as region_014, 037, 084, 105, 119, and 125 (Supplementary Table 1); however, their lack of coverage was recovered by their proximal amplicons, resulting in compensation in the whole genome, as expected.

The genotypes detected in Bangladesh from May 2020 to September 2020 were 20A, 20B, and 20F, which consisted of 25, 197, and 1 sequence, respectively. Those from March 2021 and April 2021 were 20B, 20I (alpha), 20H (beta), and 21A (delta), which consisted of 10, 6, 53, and 2 sequences, respectively. We also called 1,020 SNVs, but none of them were novel.

## 4. Discussion

In this report, we proposed two additional strategies, “the redundancy in the amplicon schemes” and “the subtraction to reduce overrepresented amplicons” to the tiling PCR-based SARS-CoV-2 whole-genome sequencing pipeline. These strategies were further validated using 304 clinical samples from patients with COVID-19 in Bangladesh.

The first strategy which we propose was introducing redundant amplicon schemes with three-group multiplex PCR. Primers designed for normal tiling PCR (e.g., Artic pipeline) have short overlaps with neighboring amplicons, enabling whole-genome coverage if all amplicons can be detected via NGS. In other words, a dropout amplicon because of the SNVs in the primer annealing sites resulted in the loss of sequence information in the region. This problem has been reported to the Artic primer scheme V3. Comparing the V3 and V4 schemes using actual clinical samples revealed that the improved primer scheme outperformed the detection of several important variants, including D950N and G142D in the spike protein [10]. Mutations continue to accumulate in the circulating SARS-CoV-2. The genetic epidemiology of the SARS-CoV-2 virus sequences in the USA demonstrated a mutation rate of 6.7 × 10^−4^ per site per year, similar to other RNA viruses [24]. It also has some hotspots under positive selection, especially in the S and N regions [24, 25]. At this rate, mutations in the primer sequence are inevitable. For example, our tiling PCR scheme (total primer sequence length: 6489 bp) will have 4.3 nucleotide changes per year. This suggests that the primer scheme should be repeatedly modified. Because of the redundant amplicon scheme in our primer design, even if one amplicon is dropped due to SNVs, the neighboring amplicon can cover the dropout.

The second strategy which we propose was the subtraction of the overrepresented amplicons from the multiplex PCR products. The counts of the untargeted amplicons, which were amplified with a primer set using biotinylated primer rate = 0 (Supplemental Table 1), increased by approximately two-fold after introducing 50 biotinylated primers. Uneven genome coverage using the tiling PCR approach has been reported in several studies and has motivated researchers to propose new primer schemes to expand sequencing coverage [9, 26]. Although these improved primer schemes outcompete the original Artic primer schemes, an inevitable amplification bias still exists. Our subtraction methods are theoretically applicable to any primer scheme. Together with the primer scheme modification due to emerging novel variants, improved primer schemes can be made more uneven using the subtraction method we introduced.

With these additional strategies combined with tiling PCR resequencing, we successfully obtained 294 whole-genome sequences from 304 Bangladeshi clinical samples from May 2019 to April 2020. They were classified into clades 20A, 20B, 20F, 20B, 20I (alpha), 20H (beta), and 21A (delta). Moreover, their detection periods were in line with global trends. Clades 20A and 20B were dominant from May to September 2020; subsequently, the first wave in Bangladesh was replaced with 20I (alpha) and 20H (beta) from March to April 2021. None of the 1,020 SNVs detected were novel, but more efforts are needed to clarify whether they diversified simultaneously or had migrated from around the world.

## 5. Conclusion

Maher et al. recently proposed a prediction method for variants of concern using sufficiently dense genomic surveillance data [27]. With the additional strategies for tiling PCR resequencing presented here, we believe that whole-genome resequencing of SARS-CoV-2 from clinical samples can be optimized and cost reduction can be achieved. These optimizations would lead to solving the regional biases in whole-genome sequence coverage out of the total number of reported cases. Furthermore, we believe that comprehensive whole-genome sequence information obtained worldwide will aid in the prediction of variants of concern.

## Data Availability

The genome sequences reported in this study are available from the GISAID and International Nucleotide Sequence Database Collaboration (INSDC) databases under accession numbers EPI_ISL_11811552 to EPI_ISL_11811841, EPI_ISL_11811846 to EPI_ISL_11811849, and PRJNA831650.

## Supplementary Materials

Supplementary Materials Supplementary Table 1: Primer sequences for tiling PCR from region_000 to region_147. The start, end, and strand columns show a 0-indexed bed formatted position in the Wuhan-Hu-1 reference SARS-CoV-2 genome.
