# Supplementary material for: COVID-19 Whole-Genome Resequencing with Redundant Tiling PCR and Subtract-Based Amplicon Normalization Successfully Characterized SARS-CoV-2 Variants in Clinical Specimens
Source: Interdiscip Perspect Infect Dis. 2022 Sep 28;2022:2109641. doi: 10.1155/2022/2109641 (PMC9534710; doi:10.1155/2022/2109641)
Supplement: Supplementary Materials — Supplementary Table 1: Primer sequences for tiling PCR from region_000 to region_147. The start, end, and strand columns show a 0-indexed bed formatted position in the Wuhan-Hu-1 reference SARS-CoV-2 genome. [file 2109641.f1.pdf]

Supplemental Table 1. Primer sequences for tiling PCR
The start, end, and strand columns show a 0-indexed bed formatted position
in the Wuhan-Hu-1 reference SARS-CoV-2 genome.

name	5' to 3' sequence	multiplex PCR group	biotinylated primer rate	Amplicon Name	start	end	strand
000F	ACCAACCAACTTTCGATCTCTTG	#1	0.99	region_000	30	53	+
000R	CGAGCATCCGAACGTTTGATG	#1	0	region_000	474	495	-
001F	ATCTAGGTTTCGTCCGGGTG	#2	0.99	region_001	230	250	+
001R	CCGTAACTATGGCCACCAGC	#2	0	region_001	655	675	-
002F	TGGCACTTGTGGCTTAGTAG	#3	0	region_002	408	428	+
002R	GTCTTTAATGCACTCAAGAGGG	#3	0	region_002	852	874	-
003F	CAGTGGCTTACCGCAAGGTTC	#1	0	region_003	608	629	+
003R	ACATTCCCCATTGAAGGTGTC	#1	0	region_003	1054	1075	-
004F	AGGGGCATACACTCGCTATG	#2	0.99	region_004	801	821	+
004R	TAACAAAATCGCCCGTCTGC	#2	0	region_004	1257	1277	-
005F	AGAGCTATGAATTGCAGACACC	#3	0.99	region_005	1004	1026	+
005R	ATAGTGCGACCACCCTTACG	#3	0	region_005	1456	1476	-
006F	CAACCAAATGTGCCTTTCAAC	#1	0	region_006	1200	1221	+
006R	ATGCCAAAATAATGGCGATCTC	#1	0	region_006	1678	1700	-
007F	ACCTGAGCATAGTCTTGCCG	#2	0	region_007	1401	1421	+
007R	AACACGAGCAGCCTCTGATG	#2	0	region_007	1874	1894	-
008F	ACTCCAAAAAGAGAAAGTCAACATC	#3	0	region_008	1623	1648	+
008R	CACCACCTGTAATGTAGGCC	#3	0	region_008	2058	2078	-
009F	GGTGCCTGGAATATTGGTGAAC	#1	0	region_009	1819	1841	+
009R	AGAATGTCTGAACACTCTCC	#1	0	region_009	2280	2300	-
010F	ATGTTCACATCTGATTTGGCTAC	#2	0	region_010	2017	2040	+
010R	TTTCTTTTGGGGCTTTTAGAGG	#2	0	region_010	2458	2480	-
011F	GCTTGTGAAATTGTCGGTGG	#3	0	region_011	2230	2250	+
011R	CCATCATATTAGGTGCAAGGGC	#3	0	region_011	2665	2687	-
012F	TTAAATCCAGAGAAGAAACTGG	#1	0	region_012	2426	2448	+
012R	GCATCTGCCACAACACAGG	#1	0	region_012	2879	2898	-
013F	TATGTTGCTCGAAATCAAAGAC	#2	0	region_013	2628	2650	+
013R	TGGCTCAAACTCTTCTTCTTCAC	#2	0	region_013	3071	3094	-
014F	ACTTAATGAGAAGTGCTCTGC	#3	0	region_014	2820	2841	+
014R	AATTGAGGTTGAACCTCAAC	#3	0	region_014	3280	3300	-
015F	TGTTCTTTCTACCCTCCAGATG	#1	0	region_015	3028	3050	+
015R	CCTGCAACACCTCCTCCATG	#1	0	region_015	3463	3483	-
016F	GGTCAACAAGACGGCAGTGAG	#2	0	region_016	3229	3250	+
016R	CGTGCTGATTAAAATTTTCATAAGC	#2	0	region_016	3664	3689	-
017F	CAGTGGTTGTTAATGCAGCC	#3	0.99	region_017	3428	3448	+
017R	AGGAATCTCAGCGATCTTTTGTTC	#3	0	region_017	3862	3886	-
018F	TCATGTTGTCGGCCCAAATG	#1	0.99	region_018	3609	3629	+
018R	AGAGTGGCAGAATCTGGATG	#1	0	region_018	4054	4074	-
019F	TCAAGCTTTTTGGAAATGAAGAG	#2	0	region_019	3826	3849	+

019R	AACCATTTAAACCCTGACCC	#2	0	region_019	4254	4274	-
020F	GTTCCTCACAGAAAACTTGTTAC	#3	0	region_020	4005	4028	+
020R	CGCTGTATAGTTGAAACTATGGC	#3	0	region_020	4453	4476	-
021F	GCCAACAGACAATTATATAACCAC	#1	0	region_021	4224	4248	+
021R	CAGAAACTGTAGCTGGCACTT	#1	0	region_021	4673	4694	-
022F	AATGCCTGTCTGTGTGGAAAC	#2	0	region_022	4428	4449	+
022R	GGAATGTGGTAGGATTACTAGTG	#2	0	region_022	4875	4898	-
023F	CACTTGGCTATGTAACACATGG	#3	0	region_023	4610	4632	+
023R	ACATCAGCTCCATCCAAATAAGTTG	#3	0	region_023	5051	5076	-
024F	CTGGACAATCTACACAACTAGG	#1	0	region_024	4814	4836	+
024R	CAGTGGCAAGATAACAGTTG	#1	0	region_024	5280	5300	-
025F	GCAAGTTGTGGACATGTCAATG	#2	0	region_025	5007	5029	+
025R	TGGCATGTTGAAACAAGTAACTC	#2	0	region_025	5460	5483	-
026F	ACATGTCAGCATTAAATCACAC	#3	0	region_026	5201	5223	+
026R	GGTGGTGCTGACATCATAAC	#3	0	region_026	5677	5697	-
027F	AGACAGTAGGTGAGTTAGGTGATG	#1	0	region_027	5423	5447	+
027R	TGGTTGTTGTGTAACTGTTTTC	#1	0	region_027	5872	5894	-
028F	AGGTGTTCAGATACCTTGTACG	#2	0	region_028	5607	5629	+
028R	AGTTAACTGGTTTAAATCATC	#2	0	region_028	6079	6100	-
029F	CGGTGCTTTACTTACAAAGTCCTC	#3	0	region_029	5811	5835	+
029R	TATACGTGGCTTTATTAGTTGC	#3	0	region_029	6253	6275	-
030F	CATATCCAAACGCAAGCTTCG	#1	0	region_030	6020	6041	+
030R	TGTCTCCTACAACTTCGGTAG	#1	0	region_030	6467	6488	-
031F	ACCTATTGTTTGGCATGTTAAC	#2	0	region_031	6228	6250	+
031R	GCTATAGTATCCCAAGGGACAC	#2	0	region_031	6659	6681	-
032F	AGAAGTAGTGGAAAATCCTACC	#3	0	region_032	6411	6433	+
032R	CGACACTCTTAACAGTATTCTTTGC	#3	0	region_032	6859	6884	-
033F	CCCTTGCTACTCATGGTTTAGC	#1	0	region_033	6626	6648	+
033R	GCCTTCTCTGTAACCAGTAC	#1	0	region_033	7052	7072	-
034F	TAGAATTAAAGCATCTATGCCG	#2	0	region_034	6828	6850	+
034R	GCAGCCAATCCAAGTACATAG	#2	0	region_034	7278	7299	-
035F	GCTGCTTTAGGTGTTTTAATGTC	#3	0	region_035	7006	7029	+
035R	CAACCGTCTACAACATGCAC	#3	0	region_035	7456	7476	-
036F	ATGGGATTTAACTGCTTTTGGC	#1	0	region_036	7209	7231	+
036R	TTAAACTGTAGTGACAAGTCTC	#1	0	region_036	7676	7698	-
037F	ATGTACATCTTCTTTGCATC	#2	0	region_037	7411	7431	+
037R	CTATAACATTAATAGGCAATGAACC	#2	0	region_037	7864	7889	-
038F	TGTGATACATTCTGTGCTGGTAG	#3	0	region_038	7627	7650	+
038R	TGTTTTGAGTTTTTCCATTGGTACG	#3	0	region_038	8073	8098	-
039F	GTTAACTTAGACAACCTGAGAG	#1	0	region_039	7828	7850	+
039R	ACAACTATCGCCAGTAACTTC	#1	0	region_039	8254	8275	-
040F	TGTTGGTGATAGTGCGGAAG	#2	0	region_040	8001	8021	+
040R	TTATTCTTTTTAGCAGCACTACG	#2	0	region_040	8452	8475	-
041F	GTTGATTCAGATGTAGAAACTAAAG	#3	0	region_041	8188	8213	+
041R	GAGTGACACCACCATCAATAGCC	#3	0	region_041	8694	8717	-
042F	AAAGTCACAACATTGCTTTG	#1	0	region_042	8378	8398	+
042R	GTAAGAAATGCAAAAAGTCACC	#1	0	region_042	8899	8921	-
043F	CTTGTGTTCCTTTTTGTTGCTGC	#2	0	region_043	8596	8619	+
043R	AACTTTCATAAGCAACAGAACC	#2	0	region_043	9085	9107	-
044F	GCCCATTGATTGCTGCAGTC	#3	0	region_044	8816	8836	+
044R	CCATCTACCACTAGTAGATACAC	#3	0	region_044	9254	9277	-

045F	TGTGTTTTGGCTGCTGAATG	#1	0.99	region_045	9001	9021	+
045R	CACCAAAAGCTCTTCTAAACCTC	#1	0	region_045	9459	9482	-
046F	GAGTACTGTAGGCACGGCAC	#2	0	region_046	9208	9228	+
046R	GTACTAAAGGTGTGAACATAACC	#2	0	region_046	9651	9674	-
047F	GCTGGTGGTATTGTAGCTATCG	#3	0	region_047	9409	9431	+
047R	TGCGTAAGAGGTAATAGCAC	#3	0	region_047	9853	9873	-
048F	CTAATGATGTTTCTTTTTTAGCAC	#1	0	region_048	9614	9638	+
048R	GCCATTTTTCTAAAACCACTCTGC	#1	0	region_048	10050	10074	-
049F	CTGTGCACCTTTTTGTTAAATAAAG	#2	0	region_049	9802	9827	+
049R	CATAGAATGTCCAATAACCCTGAG	#2	0	region_049	10276	10300	-
050F	ACAAACCTCTATCACCTCAGCTG	#3	0	region_050	10023	10046	+
050R	CCAACACTACCACATGAACC	#3	0	region_050	10480	10500	-
051F	ATGAAGATTTACTCATTCGTAAGTC	#1	0	region_051	10214	10239	+
051R	AACAGCAGCGTACAACCAAGC	#1	0	region_051	10669	10690	-
052F	ACCAATGTGCTATGAGGCCC	#2	0.99	region_052	10430	10450	+
052R	CGTCCATTCATACCATTTTGCAG	#2	0	region_052	10867	10890	-
053F	TTTGTTGACAGGCAAACAGC	#3	0.99	region_053	10606	10626	+
053R	AACAAAGACCATTGAGTACTCTG	#3	0	region_053	11050	11073	-
054F	TGCTCAAACTGGAATTGCCG	#1	0	region_054	10815	10835	+
054R	TCAACCATATCCAACCATGTC	#1	0	region_054	11256	11277	-
055F	ACACCACTGGTTGTTACTCAC	#2	0.99	region_055	11001	11022	+
055R	AGAGCCCACATGGAAATGGC	#2	0	region_055	11452	11472	-
056F	CCTGCTAGTTGGGTGATGCG	#3	0.99	region_056	11230	11250	+
056R	CACCAAGAGTCAGTCTAAAGTAGC	#3	0	region_056	11669	11693	-
057F	ATGTCTTGACACTCGTTTATAAAG	#1	0	region_057	11402	11426	+
057R	AGAGTAAGACTACTGATGTGCAC	#1	0	region_057	11862	11885	-
058F	GCTAGTTTATTGTTTCTTAGG	#2	0	region_058	11604	11625	+
058R	GTAAGGTTGCCCTGTTGTCC	#2	0	region_058	12069	12089	-
059F	TCAAAGTAGCCACTGTACAGTC	#3	0	region_059	11822	11844	+
059R	TCAGCCATCTTTTCCAACTTACG	#3	0	region_059	12259	12282	-
060F	CCATGCAGGGTGCTGTAGAC	#1	0	region_060	12023	12043	+
060R	ACCATTAGTTTGGCTGCTGTTG	#1	0	region_060	12458	12480	-
061F	GTGGCTAAATCTGAATTTGACCG	#2	0.99	region_061	12220	12243	+
061R	GCAGAATTGGCCCTTAAAGCTG	#2	0	region_061	12650	12672	-
062F	GCAAGAGATGGTTGTGTTCCC	#3	0.99	region_062	12418	12439	+
062R	TACCAGTTCCATCACTCTTAGGG	#3	0	region_062	12852	12875	-
063F	CACCTAATTTAGCATGGCCTC	#1	0	region_063	12620	12641	+
063R	AGCATCTACAGCAAAAGCACAG	#1	0	region_063	13071	13093	-
064F	TGCACTGTTATCCGATTTACAGG	#2	0.99	region_064	12810	12833	+
064R	GATCTATGTGGCAACGGCAG	#2	0	region_064	13251	13271	-
065F	AATGCAACAGAAGTGCCTGC	#3	0.99	region_065	13030	13050	+
065R	CTGCACTTACACCGCAAACC	#3	0	region_065	13469	13489	-
066F	TCCTTTGGTGGTGCATCGTG	#1	0.99	region_066	13222	13242	+
066R	TGGTAGTTAGAGAAAGTGTGTCTC	#1	0	region_066	13658	13682	-
067F	TTGTGATCAACTCCGCGAAC	#2	0	region_067	13410	13430	+
067R	CATCACAACAATTGTATGTGAC	#2	0	region_067	13878	13900	-
068F	CGCTTCCAAGAAAAGGACGAAG	#3	0	region_068	13602	13624	+
068R	ACCAGTTACCATTGAGATCTTG	#3	0	region_068	14067	14089	-
069F	TGGCAGACCTCGTCTATGC	#1	0.99	region_069	13810	13829	+
069R	AACCTCTCTTCCGTGAAGTC	#1	0	region_069	14259	14279	-
070F	GATGCCATGCGAAATGCTGG	#2	0.99	region_070	14019	14039	+

070R	AGCTCTCTGAAGTGGTATCCAG	#2	0	region_070	14470	14492	-
071F	TGTTGACACTGACTTAACAAAGCC	#3	0.99	region_071	14207	14231	+
071R	CGGGTTTGACAGTTTGAAAAGC	#3	0	region_071	14655	14677	-
072F	CCACCTACAAGTTTTGGACCAC	#1	0.99	region_072	14403	14425	+
072R	GCCACCATCGTAACAATCAAAG	#1	0	region_072	14876	14898	-
073F	CGTGCTTTTCAGTAGCTGCAC	#2	0.99	region_073	14620	14641	+
073R	TGCACTAATGGCATACTTAAGATTC	#2	0	region_073	15065	15090	-
074F	ACCAACAATGTGTGATATCAGAC	#3	0	region_074	14819	14842	+
074R	GGTGAGGGTTTTCTACATCAC	#3	0	region_074	15259	15280	-
075F	TGAGGATCAAGATGCACTTTTCG	#1	0.99	region_075	15002	15025	+
075R	AACATATAGTGAACCGCCACAC	#1	0	region_075	15443	15465	-
076F	AGCAAATTCTATGGTGGTTGGC	#2	0.99	region_076	15213	15235	+
076R	GCGTAAAACTCATTCACAAAGTCTG	#2	0	region_076	15655	15680	-
077F	TCAAGTATTGAGTGAAATGGTC	#3	0	region_077	15419	15441	+
077R	ACTAGCATTGTATGTTGAGAGC	#3	0	region_077	15877	15899	-
078F	ACACAGACTTTATGAGTGTCTC	#1	0	region_078	15611	15633	+
078R	TGAAAGACATCAGCATACTCCTG	#1	0	region_078	16062	16085	-
079F	TGAAGCAAAATGTTGGACTGAGAC	#2	0.99	region_079	15824	15848	+
079R	ACGTATGCAAGCACCACATC	#2	0	region_079	16279	16299	-
080F	GAACGGTTCGTGTCTTTAGC	#3	0.99	region_080	16008	16028	+
080R	AGCACACAATGGAAAACTAATGGG	#3	0	region_080	16467	16491	-
081F	TGAGGCTATGTACACACCGC	#1	0	region_081	16202	16222	+
081R	GTTTAAATGTCTCCTCAGTAGC	#1	0	region_081	16653	16675	-
082F	TGTGACTCAACTTTACTTAGGAGG	#2	0.99	region_082	16412	16436	+
082R	CTCGGTAAACAACAGCATCACC	#2	0	region_082	16851	16873	-
083F	AGCTTTTTGCAGCAGAAACGC	#3	0.99	region_083	16627	16648	+
083R	CTTACCAGTACCAGGTGGTCCC	#3	0	region_083	17078	17100	-
084F	AGTACACCTTTGAAAAAGGTGAC	#1	0	region_084	16825	16848	+
084R	CTGTTCTAATGTTGAATTCACTTTG	#1	0	region_084	17273	17298	-
085F	CAGATGAGTTTTCTAGCAATGTTG	#2	0	region_085	17011	17035	+
085R	TGCCCTTAGTTAGCAATGTGC	#2	0	region_085	17461	17482	-
086F	GTAGAATTATACCTGCACGTGC	#3	0	region_086	17227	17249	+
086R	GCAGATGAAACATCATGCGTG	#3	0	region_086	17675	17696	-
087F	TGTGTACATTGGCGACCCTG	#1	0	region_087	17423	17443	+
087R	GCTGTTTCAGTGGTTTGAGTG	#1	0	region_087	17873	17894	-
088F	AAGCACATAAAGACAAATCAGC	#2	0	region_088	17620	17642	+
088R	AGGATGTAACCCAGTGATTACC	#2	0	region_088	18077	18099	-
089F	ACTGTTGATTCATCACAGGGC	#3	0	region_089	17829	17850	+
089R	GCTTCTTCGCGGGTGATAAAC	#3	0	region_089	18254	18275	-
090F	GGAATGTGGCAACTTTACAAGC	#1	0.99	region_090	18019	18041	+
090R	AAATTGATCTCCAGGCGGTG	#1	0	region_090	18457	18477	-
091F	ATTATCAAGTTAATGGTTACCC	#2	0	region_091	18226	18248	+
091R	GCAGTGGAAAAGCATGTGGC	#2	0	region_091	18678	18698	-
092F	CTACAGGTTATGTTGATACACC	#3	0	region_092	18400	18422	+
092R	AAGCACTCGTGGACAGCTAG	#3	0	region_092	18876	18896	-
093F	GTATTTTGTGAAAATAGGACCTG	#1	0	region_093	18626	18649	+
093R	TGCATCATAGAACTTCCATTCTAC	#1	0	region_093	19074	19098	-
094F	TCCATGGTAATGCACATGTAGC	#2	0	region_094	18826	18848	+
094R	CACCATCACAACCAGGCAAG	#2	0	region_094	19268	19288	-
095F	TCTTCACGACATTGGTAACCC	#3	0	region_095	19022	19043	+
095R	GCACCACCTAAATTGCAACG	#3	0	region_095	19464	19484	-

096F	CCTGCTAATTCCATTGTTTGTAG	#1	0	region_096	19215	19238	+
096R	TGTTGTCCATCAAAGTGTCCC	#1	0	region_096	19655	19676	-
097F	TCATGGAAAACAAGTAGTGTCAG	#2	0	region_097	19400	19423	+
097R	GAGCATCTCTTTTGTAGTCCCAG	#2	0	region_097	19874	19897	-
098F	ACTTCAGAGTTTAGAAAATGTGGC	#3	0	region_098	19613	19637	+
098R	AGCTTGTTTGGGACCTACAG	#3	0	region_098	20080	20100	-
099F	GCAACATTAAACCAGTACCAGAGG	#1	0	region_099	19801	19825	+
099R	CCGTTCAATGAATTCATCCATAGC	#1	0	region_099	20268	20292	-
100F	TAGAAATGCCCGTAATGGTGTTC	#2	0.99	region_100	20021	20044	+
100R	ATGAACCTGTTTGCGCATCTG	#2	0	region_100	20461	20482	-
101F	GAATTTAAACCCAGGAGTCAAATGG	#3	0.99	region_101	20223	20248	+
101R	GTTGCCACGCTTGACTAGATTG	#3	0	region_101	20655	20677	-
102F	GATTTTATTCCTATGGACAGTACAG	#1	0	region_102	20418	20443	+
102R	GGTGCAACTCCTTTATCAGAACC	#1	0	region_102	20874	20897	-
103F	TGGTGTAAAGATGGCCATGTAG	#2	0	region_103	20613	20635	+
103R	CATTTTTAGTCTTAGGGTCGTAC	#2	0	region_103	21050	21073	-
104F	AACACATTAACATTAGCTGTACCC	#3	0	region_104	20820	20844	+
104R	CCAATTAAAAATGCTTCAGATGATG	#3	0	region_104	21256	21281	-
105F	TAAATGGGATCTCATTATTAGTG	#1	0	region_105	21023	21046	+
105R	TAAAATCATATCATTGATTTGACC	#1	0	region_105	21450	21474	-
106F	ACACTTCGCATGGTGGACAG	#2	0	region_106	21212	21232	+
106R	TGTCAGGGTAATAAACACCACG	#2	0	region_106	21661	21683	-
107F	AAGGGGTACTGCTGTTATGTC	#3	0.99	region_107	21419	21440	+
107R	GTAGTACCAAAAATCCAGCCTC	#3	0	region_107	21866	21888	-
108F	ACCAGAACTCAATTACCCCCTG	#1	0.99	region_108	21619	21641	+
108R	GGTCCATAAGAAAAGGCTGAGAG	#1	0	region_108	22074	22097	-
109F	GGTGTTTATTTTGCTTCCACTGAG	#2	0	region_109	21826	21850	+
109R	GCAAGTAAAGTTTGAAACCTAGTG	#2	0	region_109	22266	22290	-
110F	TGGATGGAAAGTGAGTTCAGAG	#3	0	region_110	22015	22037	+
110R	ACAGTGAAGGATTTCAACGTACAC	#3	0	region_110	22461	22485	-
111F	CTCCCTCAGGGTTTTTCGGC	#1	0	region_111	22207	22227	+
111R	CTTAAAAGTGGAAAATGATGCGG	#1	0	region_111	22673	22696	-
112F	GGAACCATTACAGATGCTGTAGAC	#2	0.99	region_112	22408	22432	+
112R	TTCCAAGCTATAACGCAGCC	#2	0	region_112	22852	22872	-
113F	CATCTGTTTATGCTTGGAACAGG	#3	0	region_113	22604	22627	+
113R	GTACTACTACTCTGTATGGTTGG	#3	0	region_113	23076	23099	-
114F	GATTATAATTATAAATTACCAGATG	#1	0	region_114	22819	22844	+
114R	GGATCACGGACAGCATCAGTAG	#1	0	region_114	23276	23298	-
115F	TGGTGTTGAAGGTTTTAATTGTTAC	#2	0	region_115	23004	23029	+
115R	AGAACCTGTAGAATAAACACGCC	#2	0	region_115	23459	23482	-
116F	AGGCACAGGTGTTCTTACTGAG	#3	0.99	region_116	23202	23224	+
116R	AGCAACTGAATTTTCTGCACC	#3	0	region_116	23659	23680	-
117F	TGCACAGAAGTCCCTGTTGC	#1	0	region_117	23410	23430	+
117R	GCTATTCCAGTTAAAGCACGG	#1	0	region_117	23853	23874	-
118F	AGTCAATCCATCATTGCCTACAC	#2	0.99	region_118	23626	23649	+
118R	CCATATTGTTTGATGAAGCCAGC	#2	0	region_118	24052	24075	-
119F	ACTGAATGCAGCAATCTTTTG	#3	0	region_119	23800	23821	+
119R	ACCTATAAGCCATTTGCATAGC	#3	0	region_119	24256	24278	-
120F	TCAACAAAGTGACACTTGCAG	#1	0.99	region_120	24029	24050	+
120R	ACACTTGAAATTGCACCAAAATTG	#1	0	region_120	24465	24489	-
121F	CACTTCTGGTTGGACCTTTGG	#2	0	region_121	24207	24228	+

121R	TTGATTGTCCAAGTACACACTC	#2	0	region_121	24652	24674	-
122F	TGGTCAACCAAAATGCACAAGC	#3	0.99	region_122	24413	24435	+
122R	TGTGTTACAAACCAGTGTGTGC	#3	0	region_122	24857	24879	-
123F	AGCTGCAGAAATCAGAGCTTC	#1	0.99	region_123	24603	24624	+
123R	GCCAGAGATGTCACCTAAATCAAC	#1	0	region_123	25051	25075	-
124F	TCCTCGTGAAGGTGTCTTTG	#2	0	region_124	24828	24848	+
124R	GCCCTTGAGACAACTACAGC	#2	0	region_124	25280	25300	-
125F	ATATTTTAAGAATCATACATCACC	#3	0	region_125	25023	25047	+
125R	CGGTATCGTTGCAGTAGCG	#3	0	region_125	25481	25500	-
126F	TGGCTTGATTGCCATAGTAATGG	#1	0.99	region_126	25227	25250	+
126R	CTTCAAGGCCAGCAGCAAC	#1	0	region_126	25680	25699	-
127F	TGGAACTGTAACTTTGAAGCAAG	#2	0	region_127	25421	25444	+
127R	TTGAAGAAGTTACACTATTGTAAGG	#2	0	region_127	25866	25891	-
128F	AGCACTCTCCAAGGGTGTTC	#3	0.99	region_128	25604	25624	+
128R	CATGTTCAACACCAGTGTCTG	#3	0	region_128	26053	26074	-
129F	GCCAACTATTTTCTTTGCTGGC	#1	0.99	region_129	25818	25840	+
129R	CGTACCTGTCTCTTCCGAAACG	#1	0	region_129	26255	26277	-
130F	CACTTCAGACTATTACCAGCTG	#2	0	region_130	26012	26034	+
130R	CGTTTAGACCAGAAGATCAGGAAC	#2	0	region_130	26451	26475	-
131F	GCACAAGCTGATGAGTACGAAC	#3	0.99	region_131	26220	26242	+
131R	CCATAACAGCCAGAGGAAAATTAAC	#3	0	region_131	26671	26696	-
132F	ACCTTCTTTTTACGTTTACTCTCG	#1	0	region_132	26402	26426	+
132R	TGGAGTGGCACGTTGAGAAG	#1	0	region_132	26876	26896	-
133F	ACAATTTGCCTATGCCAACAGG	#2	0.99	region_133	26626	26648	+
133R	AGTCACCTGCTACACGCTG	#2	0	region_133	27074	27093	-
134F	TCAGACTGTTTGCGCGTACG	#3	0	region_134	26820	26840	+
134R	CCAAATGGAAACTTTAAAAGTCCTC	#3	0	region_134	27257	27282	-
135F	TGTTGCTACATCACGAACGC	#1	0.99	region_135	27028	27048	+
135R	TTGTACCTCTAACACACTCTTGG	#1	0	region_135	27452	27475	-
136F	GTTTCATCTCGTTGACTTTCAGG	#2	0	region_136	27203	27226	+
136R	AGTTCTTGAACTTCCTCTTGTCTG	#2	0	region_136	27656	27680	-
137F	TTGGCACTGATAACACTCGC	#3	0.99	region_137	27411	27431	+
137R	TCGTTTAGGCGTGACAAGTTTC	#3	0	region_137	27869	27891	-
138F	CGTGCCAGATCAGTTTCACC	#1	0.99	region_138	27624	27644	+
138R	GCCTCATCCACGCACAATTC	#1	0	region_138	28067	28087	-
139F	TGCTTATTATCTTTTGGTTCTCAC	#2	0	region_139	27825	27849	+
139R	CAGACATTTTAGTTTGTTCGTTTAG	#2	0	region_139	28255	28280	-
140F	AATGGTATATTAGAGTAGGAGCTAG	#3	0	region_140	28023	28048	+
140R	GAACGCCTTGTCCTCGAGGG	#3	0	region_140	28470	28490	-
141F	TCATGACGTTCGTGTTGTTTTAG	#1	0.99	region_141	28225	28248	+
141R	AGGCTCCCTCAGTTGCAAC	#1	0	region_141	28669	28688	-
142F	TTGGTTCACCGCTCTCACTC	#2	0.99	region_142	28425	28445	+
142R	CCTACTGCTGCCTGGAGTTG	#2	0	region_142	28862	28882	-
143F	GCTGGACTTCCCTATGGTGC	#3	0.99	region_143	28627	28647	+
143R	CTGCCGAAAGCTTGTGTTAC	#3	0	region_143	29080	29100	-
144F	CAAGCCTCTTCTCGTTCCTC	#1	0.99	region_144	28813	28833	+
144R	GTAGGTCAACCACGTTCCCG	#1	0	region_144	29252	29272	-
145F	AAATCTGCTGCTGAGGCTTC	#2	0	region_145	29017	29037	+
145R	CCAAATCTGCAGCAGGAAGAAG	#2	0	region_145	29452	29474	-
146F	CGCATTGGCATGGAAGTCAC	#3	0	region_146	29227	29247	+
146R	CACACTGATTAAAGATTGCTATGTG	#3	0	region_146	29667	29692	-

147F	GCCTTACCGCAGAGACAGAAG	#1	0	region_147	29413	29434	+
147R	TTTTTTTTTTTTTTTGTCATTCTCC	#1	0	region_147	29860	29885	-
